# Elderly Healthcare Monitoring Using an Avatar-Based 3D Virtual Environment

**DOI:** 10.3390/ijerph10127283

**Published:** 2013-12-17

**Authors:** Matti Pouke, Jonna Häkkilä

**Affiliations:** Center for Internet Excellence, University of Oulu, Erkki Koiso-Kanttilan katu, P.O. Box 1001, 90570 Oulu, Finland; E-Mail: jonna.hakkila@cie.fi

**Keywords:** home care, elderly care, wearable sensors, 3D visualizations, avatars, information visualization, user interfaces, user studies

## Abstract

Homecare systems for elderly people are becoming increasingly important due to both economic reasons as well as patients’ preferences. Sensor-based surveillance technologies are an expected future trend, but research so far has devoted little attention to the User Interface (UI) design of such systems and the user-centric design approach. In this paper, we explore the possibilities of an avatar-based 3D visualization system, which exploits wearable sensors and human activity simulations. We present a technical prototype and the evaluation of alternative concept designs for UIs based on a 3D virtual world. The evaluation was conducted with homecare providers through focus groups and an online survey. Our results show firstly that systems taking advantage of 3D virtual world visualization techniques have potential especially due to the privacy preserving and simplified information presentation style, and secondly that simple representations and glancability should be emphasized in the design. The identified key use cases highlight that avatar-based 3D presentations can be helpful if they provide an overview as well as details on demand.

## 1. Introduction

In many Western countries, the aging of the population is a forthcoming challenge that affects several sectors in the society. In particular, the rapidly aging population highlights a concern for the health care and well-being of future generations. For instance, in Finland [1], the aging population structure will have an economic impact on the society due to increased healthcare costs. Together with potential deficit of employees [2], this leads to a trend of increasing number of elderly people living in their homes and in need of support. Therefore, the potential of smart technology should be investigated for solutions that can be harnessed to support both patients and homecare providers. Assisting elderly patients in autonomous living has the potential to reduce the strain on healthcare professionals, help elders to maintain an independent style of living, and ease the patients’ transition from home to a nursing home.

Earlier user research on elderly people has reported how the home is a place where emotional and utilitarian aspects are entwined, and which is perceived as a highly preferred place of living because of the strong affective ties, familiarity, and the notion of independence [3,4,5]. However, in the long run, the ability to maintain an autonomous style of living often calls for some level of surveillance when elderly patients in declining physical condition and memory diseases are concerned. To address this challenge, earlier work has introduced, for instance, a concept of visualizing well-being data of a distant family member through an interactive picture frame [4]. However, for the purposes of healthcare professionals, the possibility to access detailed and online data is often required. Video and microphone-based surveillance is time-consuming to observe, cumbersome, and perceived intrusive. Earlier research has shown that streaming a video from one location to another causes easily discomfort in people [6]. Privacy is a central concern when dealing with capturing technologies for patients for everyday life contexts, as discussed, for example, by Hayes and Abowd [7]. In their user research, Leonardi *et al.*, reported how elderly people were more reluctant to accept safety-related technology for places that were associated with emotional characteristics [3], which may lead to the avoidance to cover some parts of the home with surveillance systems. In order to accept assisting technologies, the patient must be able to trust his privacy is respected [8]. Indirect ways such as wearable or environmental sensors at home offer an interesting alternative to video surveillance to monitor the elderly patient’s activities and well-being [9,10]. The use of sensor networks allows the option to choose the information relevant to us, collect and display it, and opens up different possibilities for information presentation.

3D virtual environments provide a promising way to visualize elderly patients’ activities as they allow a compromise between private, non-visual and instantly understandable information provided by video surveillance, and can have the advantage of authentic-looking visualizations. Advanced presentations can be applied not only to the authentic environment and avatar design, but also to illustrate other qualities, such as dynamic security levels [11]. The Smart Condo by Boers *et al.* [12], is an example of a 3D virtual environment used to visualize information from an in-house sensor network, which can visualize the accurate location of the inhabitant as well as some activities, for example, sitting down. The work of Fleck [13] uses smart cameras for patient tracking as well as fall detection, and uses various visualization options, such as maps and even 3D environments. In case of emergency, the system can overlay a regular video feed on top of the visualization.

Telepresence has been introduced in telehealth research as a tool for medical doctors to consult patients or nurses without being physically present [14]. In our research, we take the opposing viewpoint, and study using virtual environments and telepresence to bring the elderly patient to the nurses. Previously, we have introduced a prototype which uses telepresence to visualize an elderly patient’s actions to a remote user [15]. In this paper, we focus on the early phase user-centric design of a sensor-based surveillance system utilizing aspects from telepresence and virtual world design. We are especially interested in focusing on the possibilities of utilizing 3D virtual world design together with sensor based activity recognition, and by focusing on the feedback of nursing and home healthcare professionals, charting the preferences and potential pitfalls of the approach. This paper is organized as follows. We first introduce our sensor network, activity recognition and visualization systems. Next we describe our focus group interviews and survey results. Finally, we draw conclusions of the results we found. Our work has novelty in bringing together 3D virtual world design, sensor-based activity recognition, and nursing professional’s perceptions to form a picture of potential future directions to help the researchers and designers working with elderly home care technologies.

## 2. System Overview

In this section we describe our activity data collection as well as our prototype system for elderly patient telepresence visualization. The system consists of a basic and easily transferrable sensor network capable of capturing data for activity recognition and rough location estimation [16]. Activity recognition is then combined with a visualization system, as described in our earlier work [15]. In the following, we first describe the data collection and the sensor network, the pattern recognition system we developed to detect activities from the sensor network data, and our visualization system.

### 2.1. Data Acquisition

Using our sensor network, we captured data from elderly patients at the Karpalokoti hospice in Pyhäjärvi, Finland, over a two-month period. The patients were between ages 50 and 80, all suffering from various memory diseases. We asked the patients to perform regular household activities such as dish washing and cleaning. We omitted any potentially dangerous situations, such as which might cause the patient to fall, due to the involvement of actual patients. We had to guide and observe the patients to some extent, due to the declining motor functions of some of the patients. The data acquisition sessions were recorded and the datasets acquired from the trials were annotated with the patients’ activities. Investigating the area originally from an activity-recognition and signal-processing point of view, at this point, we focused on data collection of regular household activities and thus did not gather any user requirements from medical professionals.

**Figure 1:**
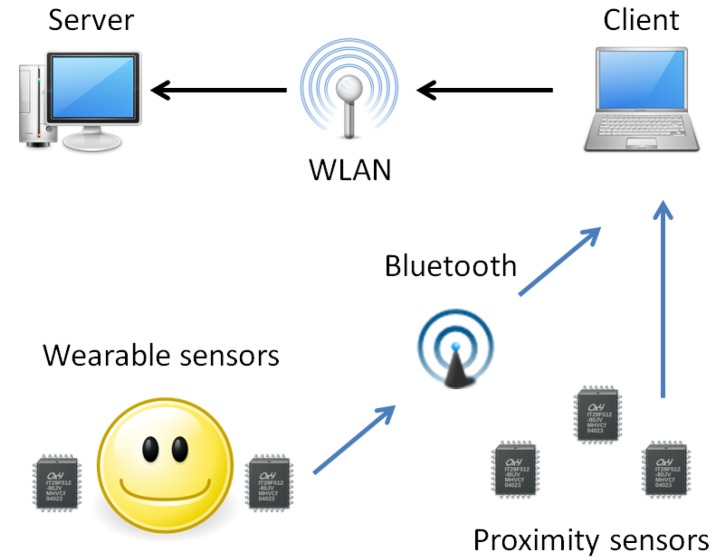
Overview of the sensor network.

Our sensor network consisted of ATR WAA-series “B-Pack” hybrid sensors, which were used as wearable sensors (WAA-006) as well as environmental sensors (WAA-001). The wearable sensors were accelerometer-gyro sensors, which were used to capture the elderly patients’ activities, while the environmental sensors were used as proximity sensors to capture each patient’s location. Each patient wore two lightweight wearable sensors in his or her wrists, and a proximity sensor master module in his or her pocket. In addition, 4–6 proximity sensors were placed at key locations in the patient’s surroundings. The sensors were capable of functioning as serial port devices through a Bluetooth connection, and we used a PDA device with client software in operating them and storing the data. If chosen, external server software could be used for data transferal through a wireless network. An overview of the sensor network can be seen in Figure 1.

The wearable sensors could sense a maximum of 5G acceleration at 500 Hz and angular accelerations of 500 deg/s for the X*-*axis and Y-axis and 300 deg/s for the Z-axis. Acceleration and angular acceleration readings were acquired at 10 ms intervals. As the patient moved throughout the surroundings, the proximity sensors transferred a signal to the client software if the master module sensor was within their preprogrammed range, here the maximum of 10 m.

### 2.2. Pattern Recognition System

Acceleration data from the wearable sensors has been successfully used in the past for recognition of various activities. The work of Bao and Intille is one of the first to describe a thorough examination of the use of various classifiers to detect activities from self-annotated acceleration data [17]. Ohmura *et al.*, describe the use of accelerometers for recognizing nursing activities [18]. Following their work, we developed a K Nearest Neighbor (K-NN) classifier to classify the activities of elderly patients and added proximity sensor-based location constraints to reduce the number of classes the classifier has to separate at a time. The K-NN classifier was suitable for our prototype as our sensor network was quite simple and a set of classes could easily be mapped to avatar animations. The pattern recognition system is described in more detail in [15].

The datasets acquired from the trials consisted of annotated acceleration and gyro timestamps at 10 ms intervals coupled with sparser proximity sensor observations. For classifying activities from the datasets, we developed a K-NN classifier using the python scripting language. For features, we first calculated the mean, standard deviation and frequency domain energy from each acceleration and gyro axis using 256 samples sliding windows with a 128 samples overlap. Moreover, correlations between left and right hand acceleration axes were calculated resulting in feature vectors with a length of 39 features in total. We then used the annotations to couple the feature vectors with their corresponding classes. At this stage of development and coming from the activity recognition background, the research did not involve medical professionals. The attempt was to annotate all activities of an elderly patient per trial, using as few action classes as possible. From this dataset, we annotated all of the patient’s activities with 18 classes.

For finding the most effective features, we performed a feature selection using the sequential forward floating search algorithm [19]. Finally we used location constraints for improving the classifier performance. The location data was used to partition actions into subsets coupled with each location to reduce the number of classes in the training space at time. With 18 classes, we reached the total classification accuracy of 80% for the dataset we chose for our visualization experiment.

### 2.3. Visualization System

For the visualization system, we created a virtual environment with the RealXtend Tundra platform [20]. The actions detected by the pattern recognition system were visualized with an animated avatar that was created with the Blender modeling toolkit. Using the classes we chose for our pattern recognition system, we aimed to represent everything the patient does with a limited number of activity animations. We categorized the actions into transition, manual actions and social/miscellaneous. The exact actions with their classification accuracies is shown in Table 1.

**Table 1:** Actions classified by the pattern recognition system and their classification accuracy as percentages.

Transition	Manual Actions	Social/Miscellaneous
Walk 81%	Open 58%	Wave hands 0%
Idle 88%	Grab 50%	Point 60%
Sit down 27%	Put down 52%	Touch head 71%
Get up 37%	Pick up (from ground) 54%	Touch abdomen 66%
	Pull 40%	
	Turn on faucet 67%	
	Turn off faucet 0%	
	Wash hands 86%	
	Sweeping motion 78%	
	Use objects 75%	

We chose to represent the 18 actions classified by the pattern recognition system with 24 avatar animations. The six additional animations were needed to have both standing and sitting animation for the *Use objects, Wave hands, Point, Touch head* and *Touch abdomen* actions. We also created *Stand* and *Sit* animations for the *Idle* class. The transition between sitting and standing animations was determined by the *Sit down* and *Get up* actions. Although only about one third of these actions were classified correctly, they could still be used rather reliably as the actual transitions consisted of multiple instances of *Sit down* and *Get up* actions. To visualize every activity of the patient, we had to choose some symbolic or higher-level animations to represent multiple actions, such as the *Use objects* gesture. The *Touch head* and *Touch abdomen* refer to any activity where the patient touches his/her head or midsection, such as when adjusting glasses or accessing pockets.

For our experiment, we created the avatar animations with a Vicon Workstation motion-capture system. The actions of an elderly patient were acted by a research assistant and the resulting motion capture data was attached to the skeletal model of our avatar. The animations were further processed in Blender and converted into the RealXtend Tundra format. In principle, a motion capture system is not required to create the animations; however animating humanoid actions from scratch requires both skill and manual work. An alternative option for animating the avatar is to use ready captured animation data, such as that in the CMU Motion Capture Database [21]. 

**Figure 2:**
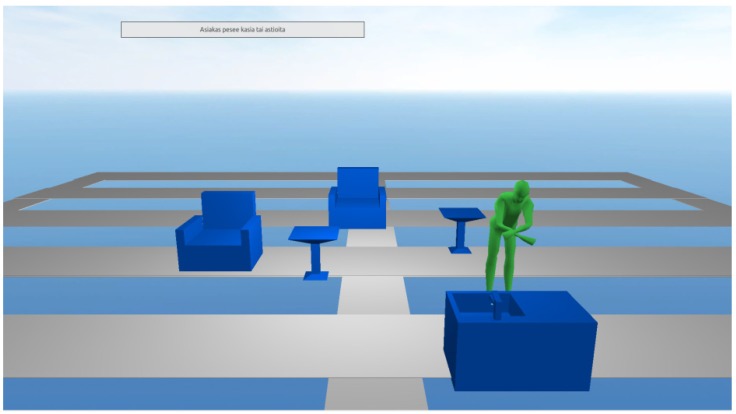
Initial visualization.

The location data from the proximity sensors was used to illustrate the patient’s location in his or her home. For this, a virtual representation of the elderly patient’s surroundings was created. Our initial virtual representation did not resemble an elderly patient’s actual surroundings, but rather attempted to describe a patient’s key locations with an arbitrary layout for home. To visualize the patient’s movements, the avatar’s location changed within the imaginary virtual representation according to five different readings from the proximity sensor data. The initial system had a single visualization to represent change in the patient’s environment; an animated water faucet displayed whether the water faucet was turned on or off. In order to help the interpretation, we also added text boxes to describe the patient’s activity as well as the state of the water faucet. The initial visualization style is shown in Figure 2.

## 3. Evaluation and Specification Gathering

Our research on activity recognition and 3D visualizations had brought us to the research question of how this approach could be utilized to design a tool for healthcare professionals, especially elderly homecare personnel. To bridge the gap between the engineering and medical domains, and to evaluate our system as well as to gather information for future development, we performed two focus group interviews with nursing staff. Following the recommendation by Razza *et al.* [22], we chose the focus group interview method to evaluate our visualization system. The method is recommended especially for research problems with open ended questions, and when the topic is wished to be discussed in a wider perspective [23]. To supplement the data, we also set up an online survey for homecare professionals. The focus group discussions were recorded on video for the data gathering. 

### 3.1. First Focus Group

The first interview (FG1) consisted of 10 nurses as well as a geriatrics medical doctor working with elderly patients in a hospice. In the interview, lasting an hour, we focused on evaluating whether the virtual environment visualization was clear and intelligible as well as assessing the usefulness of the information visualized in our system. The focus group 1 results have been partially introduced in [15] to perform a preliminary evaluation for our prototype system.

In the study, we first showed the actual virtual avatar-based system visualizing an elderly patient’s actions to the interview participants and asked for first impressions. We inquired how often they required information from a specific patient’s activities from a remote location, as well as what the activities perceived as the most important for their work were. After this, we asked the participants to fill out a written survey while showing the visualization of each activity from a video. For each activity, the participants were asked three questions: (1) the meaning of the visualization, (2) the importance of the activity and (3) the intelligibility of the animation. The first question was open-ended, whereas questions two and three were Likert-scale questions in the range of 1–5. With questions two and three, we sought to gain a rough quantification on how relevant each activity was from the nurse’s point of view, and how well the visualization succeeded in illustrating the represented activity. After going through the animations we spent the remainder of the interview discussing the potential use cases and improvements to the system. 

### 3.2. Second Focus Group

In the second focus group (FG2), we had two nurses working with elderly patient home care. In this focus group, we concentrated on acquiring the most relevant information to be visualized as well as the best visualization methods for homecare providers. Moreover, we assessed the historical information as well as automatic alarms as additional use cases of our prototype system.

**Figure 3:**
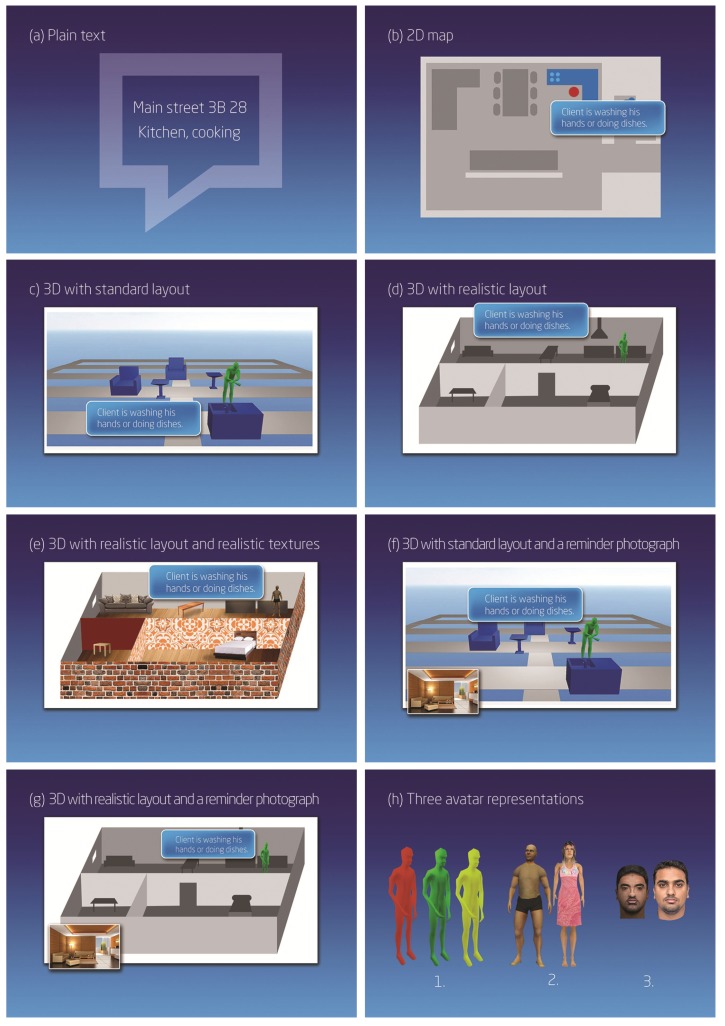
(**a**) Plain text. (**b**) 2D map. (**c**) 3D with standard layout. (**d**) 3D with realistic layout. (**e**) 3D with realistic layout and realistic textures. (**f**) 3D with standard layout and a reminder photograph. (**g**) 3D realistic layout and a reminder photograph. (**h**) Three avatar representations.

In the beginning, we asked the participants to describe the general routines and challenges in their daily work. For instance, we asked how many patients they typically worked with each day as well as what information they usually required or would find important in their work. After this, we showed a concept video introducing the animated visualization system and asked the participants to describe their first impressions. As with the first focus group, we gave the participants in the second group a written survey listing all the activities, and asked only the importance of detecting each activity in the Likert scale from 1 to 5. To investigate the homecare professionals’ perceptions of the visualization system, we created different visualization concepts with varying levels of detail; see Figure 3. The concepts were as follows:

(a) Patient’s location and activity as plain text.
(b) Patient’s location on map and activity as a text.
(c) Patient’s location in a 3D virtual environment with an imaginary layout and activity displayed with animation and text.
(d) Patient’s location in a 3D virtual environment with realistic layout and activity displayed with animation and text.
(e) Patient’s location in a 3D virtual environment with realistic textures and layout. Activity is displayed with animation and text.
(f) Patient’s location in a 3D virtual environment with an imaginary layout. Activity is displayed with animation and text. The visualization has a photograph of the patient’s house as a reminder for the nurse.
(g) Patient’s location in a 3D virtual environment with realistic layout. Activity is displayed with animation and text. The visualization has a photograph of the patient’s house as a reminder for the nurse.

Illustrations of the concepts were printed for each participant. Each alternative was discussed and the participants were asked to choose their favorite concept. Similarly, we showed three different options for the avatar representation of the patient: (1) a monochrome (different colors for different patients), (2) realistic but imaginary illustration of a person, and (3) an avatar resembling the authentic patient. The options for avatar representation are illustrated in Figure 3h. Again the participants chose their favorites and described the pros and cons they saw in each option.

### 3.3. Online Survey

In the online survey we gathered information from nurses working in private and municipal home care. Altogether 17 participants, aged 19–53, working in home care filled out the survey. Here, seven out of 17 had 1–5 years and eight out of 17 over 5 years of work experience in the field, and 14 out of 17 participants were full time homecare providers. 

The survey followed the structure and content of the second focus group. Likert scale (1–5) ratings were used to gather data about the importance of each detected patient activity and to find the preferred visualization concepts as well as avatar representations. Open-ended questions were included to gather qualitative data. In addition to the focus group procedure, the participants rated the importance of the following information about the patient’s surroundings in the Likert 1–5 scale: *Stove is turned on/off*, *Water faucet is turned on/off*, *TV is turned on/off*, *Front door is open*, *Balcony door is open.* We also asked the participants to add what information from the patient’s surroundings they would like to see visualized.

## 4. Results

This chapter summarizes the results we acquired from both focus group interviews as well as the online survey.

### 4.1. General Impressions and Suitability for User Needs

The general discussions highlighted the fast pace and high workload of homecare providers. The large number of patients and the tight schedules were seen as a challenge of the work, especially in situations where temporary changes or extra arrangements were needed, for example, due to vacations or sick leaves. The mobile system to support homecare providers was desired to deliver information of the next patient in a location-aware manner, to provide the relevant information at one glance and support manual note taking and delivery for the care professional visiting next.

**Figure 4:**
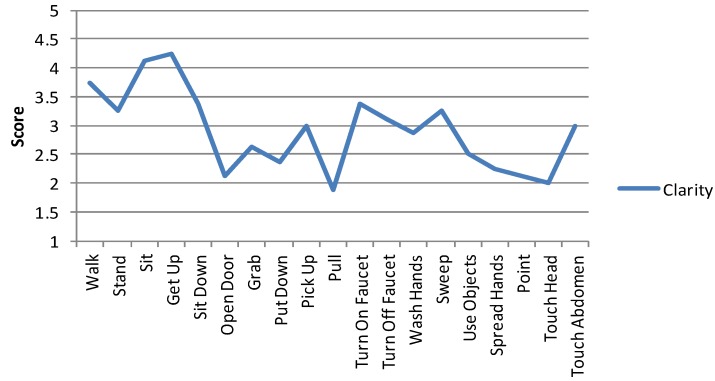
Clarity of each visualization as perceived by the participants of the first focus group.

The first impressions of the concept utilizing activity recognition and 3D virtual world design ranged from positive to very negative. While some participants saw the visualization system to be potentially helpful for nurses and patients, it was common that the system was seen at least somewhat intrusive and very intrusive by a minority of the participants. In the first interview, the participants’ concerns where alleviated towards the end of the interview session when it became clear that the system visualizes only specific, predetermined actions. Generally, the survey respondents expressed more concern about the intrusiveness of the system than the focus group participants. It was also mentioned that even if the system was helpful and useful, it might not be easy to convince the patients and their relatives to use the system. Some participants also suggested narrowing down the target group, as it was suggested that the system would be helpful with patients suffering from memory diseases, but unnecessary and too intrusive for other patients. The participants’ evaluation in focus group 1 for the clarity of each avatar animation is shown in Figure 4.

**Figure 5:**
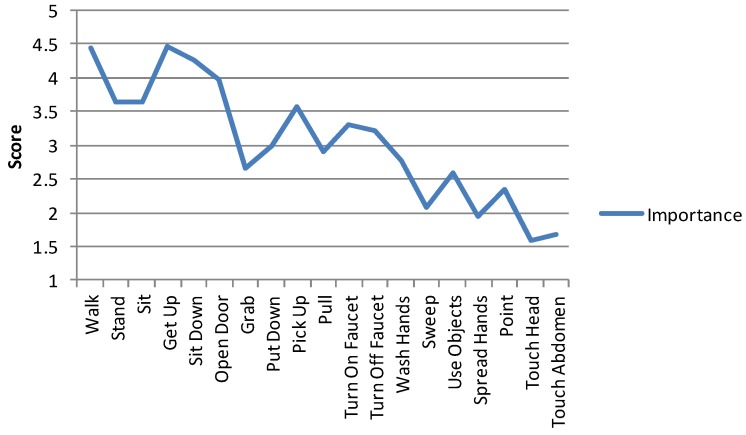
Average importance of each activity assessed by all participants.

When assessing the relevance of the visualized actions, the majority of the participants did not consider all of them relevant. The participants’ ratings for the importance of each activity is shown in Figure 5. When asked about useful activities for visualization, the most desired ones were *sleeping*, *eating*, *going to the toilet*, *falling*, and *exiting* as well as *entering the house*. Also inactivity, taking (or abusing) medicine, cooking, accessing refrigerator, movement within the apartment, going to the mailbox and walking routes outside the house were mentioned as desired information. According to the participants not only *what* but *how* the activity was performed was interesting and important especially with *walking* (FG 1, online survey). They wished the system would visualize the patient’s gait with a greater level of detail, for instance, whether the patient was walking with or without a cane, limping or using hands to keep balance.

In general, a common sentiment was that it was not necessary to see the patient’s activities in great detail. An exception was nurses, who thought detailed information of patient activities could be helpful with patients suffering from memory diseases. The key use cases identified from the findings are introduced in Table 2.

**Table 2:** Key use cases for the 3D visualization-based activity recognition system.

Use Case	Example Comments
to check how the patient fares through the day	*“The daily rhythm of a memory-impaired patient, if something happens … eating, taking medicine and other daily information like that…” (Online survey, “What information would ease your work?”).*
to help the patient to remember what he or she has or has not done	*“This could work with memory impaired patients when there’s concern if they are getting by. Quite often they cannot tell when you ask what they’ve been doing.” (Online survey).*
to verify the validity of the patient’s own reflections or narratives	“ *There is, for instance, this one patient, who claims he has done these long walks every day, and I am sure he is not able to do that. With that kind of system I could quickly check if there is any truth in there.” (FG2).*

Visualizing information of the patient’s surroundings was considered important. In general, the suggestions we made were considered important with the exception of the TV being turned on or off as shown in Table 3. In addition, the following information was desired: bathroom lights being turned on/off, general cleanliness of the apartment, any objects on the floor that might cause the patient to fall, temperature, air quality and presence of other people. 

**Table 3:** Importance of visualizing information from the patient’s surroundings.

	Very Important	Important	Moderately Important	Somewhat Important	Not Important
Stove if turned on/off	93.8% (15)	0.0% (0)	0.0% (0)	0.0% (0)	6.3% (1)
Water faucet is turned on/off	81.3% (13)	12.5% (2)	0.0% (0)	0.0% (0)	6.3% (1)
TV is turned on/off	18.8% (3)	18.8% (3)	37.5% (6)	18.8% (3)	6.3% (1)
Front door is open	87.5% (14)	6.3% (1)	0.0% (0)	0.0% (0)	6.3% (1)
Balcony door is open	56.3% (9)	31.3% (5)	6.3% (1)	0.0% (0)	6.3% (1)

### 4.2. Preferred Visualization Methods

When comparing different visualizations, Figure 3, the *plain text* and the *2D map* (Figure 3a,b ) were seen as the most desirable. From the 3D alternatives, the virtual environment with realistic layout but without textures (Figure 3d) was best received. In the second focus group interview, the participants stated that the best practice would be to show textual overview first and, after that, view more detailed information on the 3D virtual environment with realistic layout (Figure 3d,g ) if necessary. The textual presentation was commented as fast and clear to perceive, and the 3D visualization was seen potentially helpful when details, overviews, or browsing the patient activity history was needed. Several participants mentioned that realistic home layout would be useful in many cases, such as when a patient had fallen down or was moving in areas with increased risk of falling down. Majority of the nurses thought realistic textures within the virtual environment would be unnecessary or confusing. Realistic textures would increase the cognitive load when using the application and thus make its use slower. However, a photo taken from the patient’s home was seen to work well as a memory aid, and perceived as a useful addition to the plain 3D layout, Figure 3g (FG 2, online survey). Yet, showing a photo received also a criticism of presenting very, or too, intimate information (online survey, #17).

When comparing the visualizations for avatars (Figure 3h), the majority of the participants thought a monochrome avatar the most desirable choice because of its clarity; different colors could be used to distinguish different patients from each other (FG 1) or to indicate unexceptional events, for example, a detected fall (FG 2). The monochrome avatar also fit well with the mindset of making the visual design of the tool as privacy-preserving as possible. Generally, the plain visualization model was praised because of its anonymous and politically correct manner, as demonstrated, for example, in the following comment: “*Virtual avatars with the elder person’s own face sound horrid, and I don’t believe it would be accepted or understood by the patients or their families*” (online survey, #2). However, one special use case for using a realistic-looking avatar was mentioned (FG 2). The authentic looking design was seen potentially helpful in recognizing a memory-impaired patient who had left the house.

Altogether, maintaining the privacy and ability to quickly glance the most relevant information were dominating the discussions about the preferred features, and they were seen as the main strengths of the concept of using avatars and computer simulated behavior. Some individual comments were raised about the desire for visualizations that were as realistic as possible; very realistic visualizations could help in the identification of both the location and the patient. However, the participants asking for very realistic visualizations were clearly in the minority. Fast forwarding the avatar visualization was regarded as an appealing feature; the user can use it to quickly view details of activities if something unexpected, such as a fall, has been detected. However here, as well as in general, the avatar visualization was seen as a secondary view in the application UI, to be accessed when needed. The fact that the data collection method utilizes wearable sensors instead of cameras was received well. The nurses saw that information about visitors would be intruding, and were relieved when we confirmed that the system would not infer or show information related to visits: “*How about if he has a girlfriend visiting? We would not want to see that*” (FG 2).

## 5. Discussion

The research process involving nursing personnel highlighted many aspects, which we found valuable when considering the requirements for a tool for the homecare surveillance of the elderly. Preferably, the application should be quick to access, easy to glance, and show visualizations that supported in observing any exceptional events—fall, change in routines—as well as provide more details on demand. On a more focused level, the results provided valuable insight to the strengths and limitations of the concept of utilizing 3D virtual world design and computer-generated animations for visualizing the patient’s activities. 

The visually rich information presentation style was easily seen to confuse and hinder the interaction in addition to compromising privacy. From the participants’ responses it was identified that they would mostly require an overview as simple as possible and information that is more detailed as an option. An example use case for this would be the detection of a fall: the information would first be transmitted as an automated alert and shown as plain text. After this the nurse would have the option to check the patient’s location from a map, or in some cases to check the patient’s most recent activities from a quickly viewable visualization, where a 3D presentation could be used. Special cases where the value of 3D visualizations was seen as significant were: (1) checking of a memory-impaired patient’s activities during or after the day, and (2) visualizing the walking difficulties of a patient. These situations were seen as cases, where the need for detail entitles the use of 3D graphics in the visualization. 

Walking was seen as the most important activity to be detected and visualized, and especially information about different walking styles was seen highly interesting. Other often desired visualized activities were sleeping, eating, going to bathroom, falling as well as entering and exiting the house. The majority of the remote information the nurses found useful concerned the patient’s surroundings or the interaction with it. The patient’s general activities were perceived less interesting than items in the environment that might cause him or her to fall or otherwise get hurt. This is an interesting notion for the future developer of the technology, as this may require an approach different from ours, with less emphasis on wearable sensors.

The plain, monochrome design for avatars was highly preferred, and the privacy maintenance in information presentation style was a central concern. We found the preference for plain designs interesting, especially as it differs from the game domain, where 3D visualizations are currently much used. Earlier research has investigated the characteristics of a virtual character in the context of a learning task [24], and while no impact in educative outcome was detected, the using more visual complexity was found to be more fun. However, the domain of elderly home care differs radically from the infotainment applications, and we believe this is an important notion for future UI designs.

Throughout the user research it was emphasized that the person-to-person contact with the patient was important. Thus, the tool should support the professionals, not automate the work or alienate the care-takers from the patient. The respondents had an abundance of use cases for a remote surveillance system; however telepresence through a virtual environment is not necessarily in every case the best way for the system design. The virtual environment visualizations can be unnecessarily complex and seem realistic enough for some people to feel very intrusive. In addition to those of the nurses, also the patients’ and their families’ opinions can affect the acceptance of the technology. 

This research represents the first phase of our research involving the nursing personnel, and in future studies we wish to conduct participatory design sessions and evaluations with more advanced prototypes and designs. We acknowledge that our research is limited by the sample size and cultural settings of the study. Moreover, the early state of our prototype system prevented us to try out our concept design in the wild in authentic homecare context*.* Yet, we believe that our research has resulted interesting insight to the problem area. The key findings were raised repeatedly in the collected data, and we believe that they can help in design decisions when developing tools for future homecare. 

## 6. Conclusions

In this paper, we have addressed the challenges of designing surveillance systems for elderly people living at home. We have investigated the possibilities of a sensor based, wearable activity-detection system, which relies on simulation models and 3D environments in information visualization. Our concept design is based on our prototype system, where we have achieved the detection rate of roughly 80% for 19 everyday life activities. This paper focuses on evaluating different concept level visualizations with elderly homecare professionals. Our paper contributes in providing a nursing personnel’s viewpoint and background information when designing future surveillance systems for elderly homecare.

Our findings reveal that our approach of utilizing 3D models and computer-generated simulations in information presentation has both strengths and weaknesses. The advantages include especially the ability to maintain the patient’s privacy and to check details and browse temporal content when evidence based information is being sought. As a disadvantage, 3D virtual world-based visualization can be unnecessarily complex and intrusive. Complex visualizations with unnecessary visual details were seen to hinder and slow down the interaction, and preference was given to simple visual presentations. While the 3D visualization technique has its advantages, the user interfaces should carefully be designed to support the work in optimal ways, and should rather be used as an option. The 3D visualization technique was speculated to be most useful in specific scenarios where extra detail was needed. While regular text or map visualizations were perceived to function well most of the time, 3D visualizations were considered useful for providing additional insight in extraordinary situations. Based on these findings, we conclude that 3D visualizations would be most useful when used as an additional detail layer according to the need.

The work of home care professionals was often fast paced and covered a large number of patients. Consequently, the design of mobile computing tools should emphasize clear information presentation style. The capability to quickly check the activities, for example, from visual playback was appreciated. The feature would be valuable when verifying the patient’s verbal reports or confirming the rest and sleep routines. Moreover, it can provide a way to check details of what happened, for example, before an accident of fall.

In future studies, in addition to leveraging our findings in further prototypes, we seek to evaluate the feasibility of 3D visualizations while comparing them to more traditional visualizations. In the next phase, our plan is to develop more advanced prototypes and application UIs, which can be evaluated and iterated further through participatory design. These prototypes should seek to implement the features requested by the focus group participants and, on the technology side, improve the sensor network and associated pattern recognition systems accordingly.
